# Germacrene A synthase in yarrow *(Achillea millefolium*) is an enzyme with mixed substrate specificity: gene cloning, functional characterization and expression analysis

**DOI:** 10.3389/fpls.2015.00111

**Published:** 2015-03-03

**Authors:** Leila Pazouki, Hamid R. Memari, Astrid Kännaste, Rudolf Bichele, Ülo Niinemets

**Affiliations:** ^1^Institute of Agricultural and Environmental Sciences, Estonian University of Life SciencesTartu, Estonia; ^2^Biotechnology and Life Science Center and School of Agriculture, Shahid Chamran UniversityAhvaz, Iran; ^3^Molecular Pathology, Institute of Biomedicine and Translational Medicine, University of TartuTartu, Estonia; ^4^Estonian Academy of SciencesTallinn, Estonia

**Keywords:** *Achillea millefolium*, cytosolic terpene synthesis, enzyme assay, gene expression, germacrene A synthase gene, mixed substrate specificity, monoterpenes, sesquiterpenes

## Abstract

Terpenoid synthases constitute a highly diverse gene family producing a wide range of cyclic and acyclic molecules consisting of isoprene (C5) residues. Often a single terpene synthase produces a spectrum of molecules of given chain length, but some terpene synthases can use multiple substrates, producing products of different chain length. Only a few such enzymes has been characterized, but the capacity for multiple-substrate use can be more widespread than previously thought. Here we focused on germacrene A synthase (GAS) that is a key cytosolic enzyme in the sesquiterpene lactone biosynthesis pathway in the important medicinal plant *Achillea millefolium (AmGAS)*. The full length encoding gene was heterologously expressed in *Escherichia coli* BL21 (DE3), functionally characterized, and its *in vivo* expression was analyzed. The recombinant protein catalyzed formation of germacrene A with the C15 substrate farnesyl diphosphate (FDP), while acyclic monoterpenes were formed with the C10 substrate geranyl diphosphate (GDP) and cyclic monoterpenes with the C10 substrate neryl diphosphate (NDP). Although monoterpene synthesis has been assumed to be confined exclusively to plastids, AmGAS can potentially synthesize monoterpenes in cytosol when GDP or NDP become available. AmGAS enzyme had high homology with *GAS* sequences from other Asteraceae species, suggesting that multi-substrate use can be more widespread among germacrene A synthases than previously thought. Expression studies indicated that *AmGAS* was expressed in both autotrophic and heterotrophic plant compartments with the highest expression levels in leaves and flowers. To our knowledge, this is the first report on the cloning and characterization of germacrene A synthase coding gene in *A. millefolium*, and multi-substrate use of GAS enzymes.

## Introduction

A large variety of volatile organic compounds (VOCs) are synthesized and released into the environment by plants (Pichersky and Gershenzon, 2002). Although VOCs include a wide range of hydrocarbons and oxygenated hydrocarbons, terpenoids consisting of isoprene, monoterpenes and sesquiterpenes constitute the largest class of VOCs in ambient atmosphere (Guenther et al., 1995, 2000; Fineschi et al., 2013). Overall, over 60,000 terpenes and derivatives are found in nature (Cheng et al., 2007; Bohlmann and Keeling, 2008). Terpenoids are synthesized by a variety of terpenoid synthases that are characterized by variation in substrate and product specificity and expression level in different tissues (Christianson, 2006, 2008; Cheng et al., 2007; Bohlmann and Keeling, 2008; Nagegowda, 2010; Rajabi et al., 2013). During recent decades, there has been major progress in identification and functional characterization of volatile terpenoid biosynthesis genes, enzymes and in metabolic engineering of terpenoid synthesis, and this has contributed greatly to improved understanding of basic mechanisms and variability of terpenoid biosynthesis (Keeling and Bohlmann, 2006; Bohlmann and Keeling, 2008; Degenhardt et al., 2009; Nagegowda, 2010; Chen et al., 2011; Rajabi et al., 2013). However, we still lack information of gene structure, expression regulation and catalysis mechanisms for a large number of biologically and economically important terpenoid synthases.

Sesquiterpenes are synthesized by sesquiterpene synthases and play a variety of ecological roles in higher plants. Many sesquiterpenes are volatile compounds that are commonly emitted from flowers serving as attractants to pollinators (Morse et al., 2012), but also as repellents against nectar thieves (Junker and Bluethgen, 2008). In addition, sesquiterpene emissions from leaves of several plant species play important roles in direct and indirect chemical defense against pathogens and herbivores (Schnee et al., 2002; Cheng et al., 2007; Chappell and Coates, 2010). They can serve both as repellents (Huang et al., 2012; Scala et al., 2013) or as attractants of herbivore predators and parasitoids (Schnee et al., 2002). Sesquiterpenes are also synthesized and accumulated in underground organs like rhizomes and roots (De Kraker et al., 1998; Kovacevic et al., 2002; Rasmann et al., 2005) where they participate in attracting nematode predators (Rasmann et al., 2005).

Sesquiterpenes, including germacrenes, are particularly abundant in the Asteraceae family. In several species belonging to Asteraceae, germacrenes fulfill a central role in the formation of different sesquiterpene derivatives, in particular, sesquiterpene lactones (Adio, 2009a). Sesquiterpene lactones exhibit important pharmacological, physiological and ecological features. For example, artemisinin is an antimalarial sesquiterpene lactone produced by *Artemisia annua* (Ro et al., 2006; Keasling, 2012; Paddon et al., 2013). Production of this important pharmaceutical has recently been commercialized in heterologous systems (Ro et al., 2006; Keasling, 2012; Paddon et al., 2013). Sesquiterpene lactones also have antimigraine, antifungal and antibacterial properties and can protect against pests and herbivores (Picman, 1986). Recently, some important biofuels have been developed from sesquiterpene derivatives (Mcandrew et al., 2011).

Among Asteraceae, the genus *Achillea* contains over 100 herbaceous species spread throughout the northern hemisphere. The aerial parts of species from this genus are widely used in herbal medicine for preparation of infusion with antiphlogistic and spasmolytic activity (Nemeth and Bernath, 2008). Different groups of sesquiterpene lactones have been reported from this genus, eudesmanolides, and guaianolides being the most common (Si et al., 2006). Aerial parts of *Achillea millefolium* L., one of the most wide-spread and important medicinal species, have long been used as a drug in traditional and modern medicine and in herbal teas, curing inflammation and gastrointestinal spasms (Chandler et al., 1982). Sesquiterpene lactones have been identified as major compounds in *A. millefolium* (Montsko et al., 2008) and a number of germacranolides and guaianolides has already been identified in this species (Glasl et al., 2002). Some other sesquiterpene lactones such as 8-α-angeloxy–artabsin, 8-α-tigloxy–artabsin, 8-α-angeloxy-3-oxa-artabsin, 8-α-tigloxy–3-oxa-artabsin, 8-desacetyl-matricarin and santonin have also been detected in *A. millefolium* by LC-MS (Montsko et al., 2008).

Sesquiterpene lactones are derived from sesquiterpene (+)-germacrene A in many plant species, including Asteraceae (De Kraker et al., 2001). Among germacrene A derived lactones, there are a number of pharmaceutically important compounds such as parthenolide in feverfew (*Tanacetum parthenium*) (Majdi et al., 2011), tenulin (yellow sneezeweed, *Helenium amarum*) and helenalin (sneezeweed, *Helenium autumnale*) (Bouwmeester et al., 2002). Furthermore, germacrene A itself and in particular its rearrangement product β-elemene have been shown to possess anticancer activity (Adio, 2009b).

Germacrene A is formed from farnesyl diphosphate (FDP) by germacrene A synthase (GAS) (De Kraker et al., 1998). The gene structure of *GAS* and the enzyme functional activity have been studied only in a few species (De Kraker et al., 1998; Bouwmeester et al., 2002; Majdi et al., 2011), and there is thus, limited information on biological variation in sequence structure, expression and catalysis. Furthermore, there is overall limited information on key sesquiterpene synthases involved in physiological processes, in particular, on factors determining the substrate profiles of these enzymes. Recently, it has been demonstrated that some sesquiterpene synthases can catalyze both formation of sesquiterpenes with C15 substrate and monoterpenes with C10 substrate (Davidovich-Rikanati et al., 2008; Gutensohn et al., 2013; Rajabi et al., 2013), but it is unclear how general this finding is. The synthesis of hemiterpenes (C5), monoterpenes (C10) and diterpenes (C20) has been thought to occur in plastids, while that of sesquiterpenes (C15) and triterpenes (C30) to occur in cytosol (Cheng et al., 2007; Davidovich-Rikanati et al., 2008; Gutensohn et al., 2013; Rajabi et al., 2013). However, recent evidence suggests that multiple-substrate sesquiterpene synthases can catalyze monoterpene formation in cytosol (Davidovich-Rikanati et al., 2008; Gutensohn et al., 2013), providing a hugely exciting way of regulation of compound profiles, sesqui- vs. monoterpenes, by alterations in cytosolic pool sizes of different substrates. Alteration of product profiles as the result of substrate changes can have important consequences for terpenoid accumulation in aromatic species lacking specialized storage structures. Use of multiple substrates in functional characterization of terpenoid synthases is by far not a routine procedure (Davidovich-Rikanati et al., 2008; Gutensohn et al., 2013; Rajabi et al., 2013), and there is, as yet, no evidence of monoterpene synthase activity for GAS enzymes.

To gain insight into terpenoid synthesis in *A. millefolium* and its regulation, the objectives of this study were molecular identification and functional characterization of germacrene A synthase in *A. millefolium* and quantification of germacrene A synthase gene expression in different tissues. The results of this study demonstrate that *A. millefolium* GAS enzyme is a multi-substrate enzyme catalyzing formation of germacrene A, but also acyclic and cyclic monoterpenes depending on the substrate available.

## Materials and methods

### Plant material

Field-grown yarrow (*A. millefolium*) plants of local genotype (Tartu, Estonia, 58°23′N, 27°05′E) were transplanted in clay pots of 3 L and grown under controlled conditions in a growth chamber (16 h day length and day/night temperature of 25/18°C, incident quantum flux density of 400 μmol m^−2^ s^−1^). Flowers, leaves, roots, rhizomes and stems were collected and immediately frozen in liquid nitrogen and stored at −80°C for gene expression analysis (three biological replicates for each tissue were used). Fresh yarrow flowers (4 g dry weight) and leaves (7 g dry weight) were harvested for the analysis of volatiles from the field in August 2013.

Germacrene A has been previously found to accumulate in chicory (*Cichorium intybus* L.) roots (De Kraker et al., 1998). Due to lack of germacrene A as a reference standard we also analyzed chicory roots to get a baseline estimate of the sensitivity of germacrene A detection by our laboratory setup. Fresh roots of chicory were harvested in the field in October 2013. In the laboratory, chicory roots were cleaned and stored at −80°C until chemical analyses.

### *In vivo* sampling of volatiles for gas-chromatograph mass-spectrometer (GC-MS) analyses

Fresh flowers and leaves of *A. millefolium* were enclosed in a 35 × 43 cm ovenproof polyethylene terephthalate bag (PEFT) (Stewart-Jones and Poppy, 2006; Niinemets et al., 2011), and conditioned at 30°C for 3–4 h under a light intensity of 1000 μmol m^−2^ s^−1^. A solid-phase microextraction (SPME) fiber of 65 μm of polydimethylsiloxane/divinylbenzene (PDMS/DVB, Supelco, Bellefonte, PA, USA) was then inserted in the headspace for sampling of volatiles. Sampling with SPME has been previously demonstrated to provide excellent means to assess the composition of volatiles in *A. millefolium* (Cornu et al., 2001). After 20 min of sampling, the fiber was removed from the bag and immediately transferred to the injection port of the gas-chromatograph mass-spectrometer (GC-MS; GC 2010 and QP 2010 Plus, Shimadzu Corporation, Kyoto, Japan). Three biological replicates were used for collection of volatiles.

Roots of chicory (*C. intybus*) (1 g dry mass) were homogenized, and the homogenate was inserted in an ovenproof 10 × 15 cm PEFT bag for 1 h at 30°C. The SPME fiber was inserted into the headspace for 20 min and then immediately transferred into the injector of the GC-MS.

Separate samples were used to estimate dry (oven-drying at 70°C to a constant mass) to fresh mass ratio of each analyzed plant fraction.

### GC-MS analysis

Volatiles collected onto SPME-fiber were analyzed using the Shimadzu GC-MS system. A GC column ZB5-MS (0.25 mm i.d. × 30 m, 0.25 μm film Zebron, Phenomenex, Torrance, CA, USA) was employed for separating the volatiles using the following temperature program: 40°C for 3 min, ramp of 7°C min^−1^ to 220°C followed by a 5 min hold. When developing the GC-MS protocol, various injector temperatures between 215°C and 120°C were tested. As demonstrated previously, high temperature caused the bulk of germacrene A to be converted into β-elemene through Cope rearrangement (De Kraker et al., 1998; Adio, 2009a). However, too low temperatures resulted in incomplete desorption. Thus, throughout the study we used an optimized GC-MS injector temperature of 150°C.

The mass spectrometer was operated in electron-impact mode at 70 eV and in the scan range *m/z* of 30–400 amu. The transfer line temperature was set at 240°C and ion-source temperature at 150°C. Terpenes were identified by comparing their mass spectra and retention indices (RIs) for ZB5-MS to the spectra available in the NIST library (National Institute of Standards and Technology) and using a catalog of essential oil components (Adams, 2001). Commercially available reference compounds were purchased from Sigma-Aldrich (St. Louis, MO, USA) at the highest purity available (>98%). Based on serial dilution of standards, we estimated that the analytical detection threshold for sesquiterpenes in headspace was better than 0.1 nmol ml^−1^, and the minimum emission rate that could be detected was lower than 2 ng g^−1^ DW h^−1^ (ca. 50-fold lower than the typical detection threshold of ca. 0.1 μ g g^−1^ DW h^−1^). Thus, the analytical precision of our setup was suitable to detect emissions through the high to low emission range.

### Amplification of germacrene A synthase gene and rapid amplification of cDNA ends (RACE-PCR)

Total RNA was extracted from different tissues using RNeasy Mini Kit (Qiagen, Venlo, The Netherlands). The RNA was checked by agarose gel electrophoresis (Sigma-Aldrich, St. Louis, MO, USA) and the quality was evaluated by Bioanalyzer 2100 (Agilent, Santa Clara, CA, USA). The reverse transcription reaction for cDNA synthesis was carried out using iScript cDNA Synthesis Kit (Bio-Rad, Hercules, CA, USA). Based on a comparison of sequences of germacrene A synthases, three degenerate primer pairs GAS1, GAS2, and GAS3 (Table 1), were designed for six conserved regions and the polymerase chain reaction (PCR) was performed. The amplicons were either run on agarose gel, or checked by agarose gel electrophoresis and they showed fragments of approximately 894 bp, 567 bp and 402 bp for GAS1, GAS2 and GAS3 fragments, respectively. The PCR product from GAS2 (green boxes in **Figure 2**) was purified and inserted into a pTZ57R/T vector and transformed to *E. coli* using InsTAclone PCR Cloning Kit (Thermo Scientific, Pittsburgh, PA, USA). Fourteen individual transformants were bidirectionally sequenced and finally assembled by MEGA 5 software (Tamura et al., 2011). Two rounds of 5′ and 3′ RACE were done using the 5′/3′ RACE Kit (Roche Diagnostics, Indianapolis, IN, USA) according to the manufacturer's protocol. The single strand cDNA for 3′ and 5′ ends were synthesized from 1000 ng of total RNA extracted from yarrow flowers. Based on the partial coding sequence (CDS) of *AmGAS*, nested primers were designed (Table 1, RACE-GAS). PCR was conducted as specified in the previous section and the PCR products were sequenced, assembled by MEGA 5 software and full length cDNA of *A. millefolium* germacrene A synthase (*AmGAS*) was established (Tamura et al., 2011). The full length sequence of *A. millefolium* germacrene A synthase (*AmGAS*) was registered in GenBank, http://www.ncbi.nlm.nih.gov/ with accession number KC145534 and integrated into UniProtKB/TrEMBL, http://www.uniprot.org/ with accession number L7XCQ7.

**Table 1:** **Sequence of primers used in this study**.

**Primer name^a^**	**Sequence (5′—3′)**	**Product size (bp)**
GAS1	GAS1-F: AGACCATTYCATCARGGGATGC	894
	GAS1-R: CTCGTTDATATCYTTCCAYGCATTYTC	
GAS2	GAS2-F: TTYCCTCCTTCDGTATGGGGTGA	567
	GAS2-R: TGG CAT CCC TTG ATG RAA TGG TC	
GAS3	GAS3-F: GCATCATTTCCDGAGTAYATGAAG	402
	GAS3-R: CCTGTAYACVACATCTATCATTC	
RACE-GAS	RACE-GAS-F: GATGAAGCCTCGGTTTTCATCGAAGG	1150
	RACE-GAS-R: CACAAG GTAGTTTGTAACCAAGGTGTCG	
AmGAS	AmGAS-F: AATTCCATGGCAGCGGTTCAAGCTACTACTGGTATC	1680
	*NcoI*^b^	
	AmGAS-R: GATGCTCGAGTTAGTGGTGGTGGTGGTGGTGCACGGGTAGAGAATCCACAAAC	
	*XhoI*^c^	
GAPDH	GAPDH-F: ACTGGTGTCTTCACTGACAAGGA	135
	GAPDH-R: GTA TCCCCATTCGTTGTCGTACCA	
β-actin	β-actin-F: ATGGAGAAGATCTGGCATCA	130
	β-actin-R: GGAAGCTGCTGGTATTCATGAGAC	
RtGAS	RtGAS-F: CTCGGGTACTTTCAAGGAATCC	123
	RtGAS-R: CTTCGATGAAAA CCGAGGCTTC	

a GAS1, GAS2, GAS3, degenerate primers for germacrene A synthase (GAS); RACE-GAS, GAS nested primer for rapid amplification of cDNA based on partial sequence of AmGAS; AmGAS, Achillea millefolium germacrene A synthase; GAPDH, glyceraldehyde 3-phosphate dehydrogenase (GenBank asscession number KF286432); β-actin (GenBank accession number JX679606); RtGAS, real time PCR primer for AmGAS.

b,c Sequence of restriction enzymes.

### Phylogenetic tree of germacrene A synthases and multiple sequence alignment

**Figure 1:**
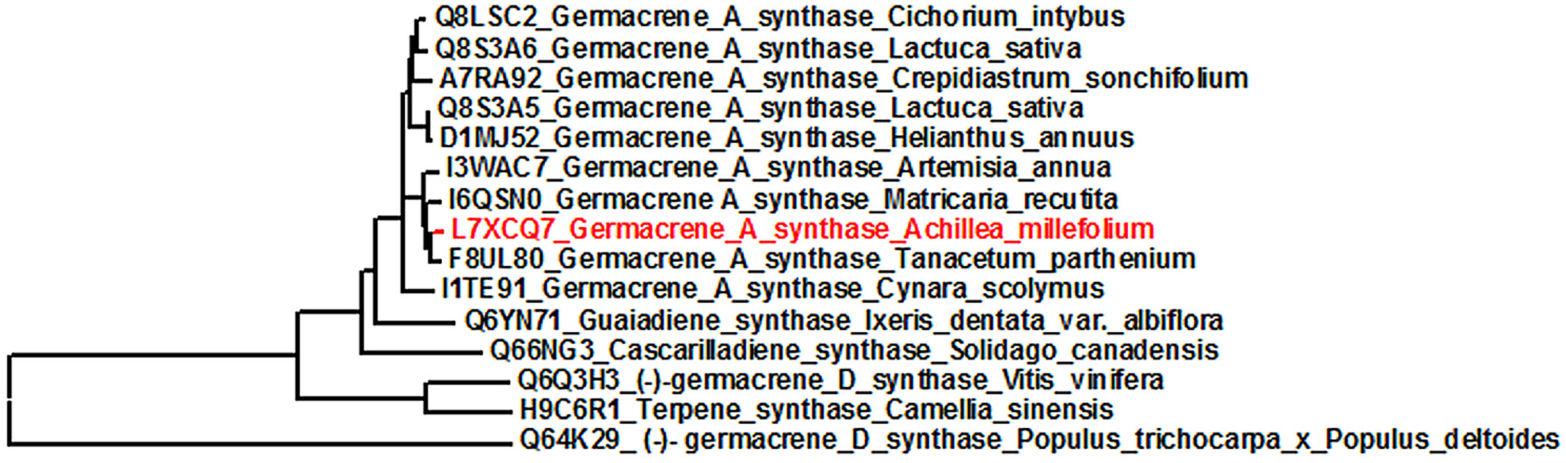
**Phylogenetic tree analysis**. Tree was built on the basis of germacrene A synthase (*GAS*) gene in *A. millefolium* with other germacrene A synthase genes, and two sesquiterpene synthases from the family Asteraceae and terpene synthases from other more distant families included as an outgroup (*Vitis vinifera*, *Camellia sinensis* and *Populus trichocarpa* x *P. deltoides*).

**Figure 2:**
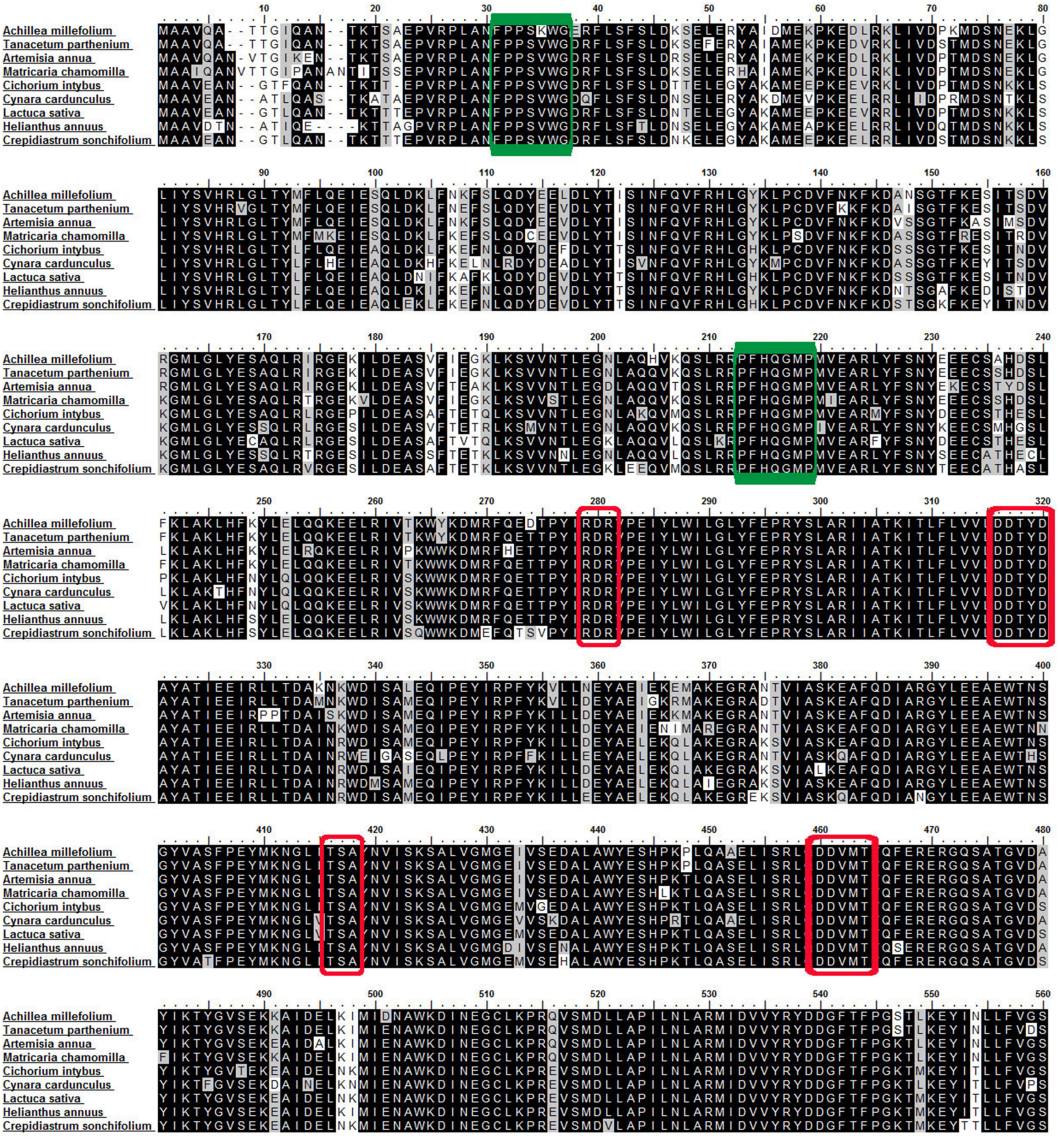
**Multiple sequence alignment of germacrene A synthases**. Amino acid sequence alignment of AmGAS with other germacrene A synthases from the Asteraceae family. The green boxes show position of degenerate *GAS* primers used. The red boxes highlight conserved sequences (see Discussion). The alignment was conducted with BioEdit software ver. 7 (http://www.ebi.ac.uk/Tools/clustalw2/index.html).

Germacrene A cDNA from *A. millefolium* was translated to the corresponding amino acid sequence and aligned and compared with other related terpenoid synthase gene sequences for Asteraceae and *GAS*-like sequences in phylogenetically distant angiosperms in UniProtKB/TrEMBL, http://www.uniprot.org/. A phylogenetic tree (Figure 1) was generated by MEGA 5 software using the UPGMA method (Tamura et al., 2011). Multiple sequence alignment was done to visualize conserved sequences among germacrene A amino acid sequences in Asteraceae (Figure 2) with BioEdit software ver. 7 (http://www.ebi.ac.uk/Tools/clustalw2/index.html).

### Gene cloning and construction of the expression vector

The *AmGAS* gene was amplified using specific primers containing restriction sites. Primer pairs were designed for amplification and cloning of full length *AmGAS* (~1700) gene (Table 1). Plasmid pET-26b (+) (Novagen, Madison, WI, USA) was used as the expression vector for *AmGAS*. The *AmGAS* PCR product and pET-26b (+) expression vector were digested with *NcoI* and *XhoI* restriction enzymes according to the manufacturer's protocol (New England Biolabs, Ipswich, MA, USA). The digested fragments were gel-purified and then *AmGAS* fragment was cloned into pET-26b (+) expression vector and transformed to *E. coli* BL21 (Novagen) using the calcium chloride transformation method (Sambrook and Russell, 2001). The lines were screened by culturing on a LB agar medium containing 50 μg ml^−1^ kanamycin. The obtained colonies were used in a colony PCR assay using *AmGAS*-specific primers (Table 1). The plasmids from positive colonies according to the PCR screening were digested with the same restriction enzymes used for cloning (*NcoI* and *XhoI*). The recombinant strains were selected and expression plasmid confirmed by sequencing analysis.

### Expression of recombinant germacrene A synthase in *Escherichia coli*

A recombinant strain colony containing *AmGAS* gene was used in the protein expression experiment. A *E. coli* BL21 (DE3) strain containing pET-26b (+) vector was used as a control. To induce expression, isopropyl-β-D-thiogalactoside (IPTG) was added to a final concentration of 1 mM to cultures with OD_600_ (optical density at a wavelength of 600 nm) of 0.4. Cultures were incubated at 37°C for 2, 4, and 6 h.

### Electrophoretic analysis of recombinant germacrene A synthase

Expression of *AmGAS* was confirmed by SDS-PAGE and western blotting. Bacterial samples were collected before (control) and after induction, and lysed in the sample buffer (100 mM Tris-HCl, pH = 8, 20% glycerol, 4% sodium dodecyl sulfate (SDS), 2% β-mercaptoethanol, 0.2% boromo phenol blue). BlueStar prestained protein marker (Nippon Genetics Europe, Düren, Germany) was used as a size standard. Samples were analyzed by 12% sodium dodecyl sulfate–polyacrylamide gel electrophoresis (SDS-PAGE) on a Bio-Rad Mini Protean electrophoresis unit. Protein bands were visualized by staining with Coomassie brilliant blue R-250.

Proteins were also transferred to a nitrocellulose membrane (Bio-Rad), for western blot analysis with 3, 3′-diaminobenzidine (DAB) liquid substrate system tetrahydrochloride (Sigma–Aldrich). The recombinant AmGAS has a His-tag in the C-terminus, and thus, the expression can be detected by anti His-tag peroxidase.

### Functional characterization of germacrene A synthase

For *in vitro* germacrene A production, cultures of pET-26b (+) plus *AmGAS* were grown to an OD_600_ of 0.4, induced using IPTG (1 mM) and grown for 6 h. These cultures were pelleted by centrifugation for 5 min at 10,000 rpm and kept at −80°C. Frozen pellets were suspended in 1 mL of assay buffer selected for optimum pH 7 and ionic strength (25 mM Hepes pH 7.2, 100 mM KCl, 10 mM MnCl_2_, 10% glycerol, and 5 mM DTT) (Fischbach et al., 2001; Reiling et al., 2004; Rajabi et al., 2013) and lysed on ice by sonication for 1 min. Lysates were centrifuged at 17,530 RCF for 30 min at 4°C. A 200 μL of the supernatant was added into 800 μL of assay buffer in a 4 mL serum vial. We tested the use of C15 substrate farnesyl diphosphate (C15) and *cis*-configured C10 substrate neryl diphosphate (NDP) and *trans*-configured C10 substrate geranyl diphosphate (GDP). Four serum vials were considered for different substrates and then 2 μL of (1 mg mL^−1^ aqueous solution) substrate (either FDP, GDP, mixture of FDP and GDP, mixture of FDP and NDP, mixture of GDP, NDP or NDP (Echelon Biosciences, Salt Lake City, UT, USA) was added to vials to start the reaction, and the vials were sealed. The vials were kept at 30°C for 50 min until collection of volatiles from the headspace.

To collect volatiles, a SPME fiber was inserted through the cap of the vial into the headspace for 1 min. After removal from the headspace, the fiber was transferred immediately into the injector of GC-MS and the analysis of volatiles was carried out as detailed in the section *GC-MS analyses*.

### Isolation of housekeeping genes and primer design for real-time PCR

Real-time PCR measurements for expression of genes of interest need to be normalized with respect to the housekeeping genes that are constitutively expressed in nearly all tissues and all physiological stages of an organism (Nicot et al., 2005; Maloukh et al., 2009). Two housekeeping genes, β-actin and glyceraldehyde 3-phosphate dehydrogenase (GAPDH), were used for this study. Based on comparison of sequences of β-actin and GAPDH of related species, two primer pairs were designed on the basis of conserved regions (Table 1). Two PCR products were purified and inserted in pTZ57R/T vector and transformed to *E. coli* using InsTAclone PCR Cloning Kit. Four individual transformants were sequenced and assembled by MEGA 5 software. The sequences of β-actin and GAPDH were registered in GenBank, http://www.ncbi.nlm.nih.gov/, with accession number JX679606.1 and KF286432, respectively.

The real-time PCR primers for *AmGAS* (Table 1, RtGAS) and housekeeping genes (Table 1) were designed on the basis of their sequences through GenScript real-time PCR primer design, https://www.genscript.com/ssl-bin/app/primer.

### Gene expression analysis of germacrene A synthase in different tissues

RNA was extracted with three independent biological replicates from different tissues (flowers at different stages of development, leaf, stem, rhizome, and root) and quantified using a BioPhotometer plus (Eppendorf, Hamburg, Germany). First-strand cDNA was synthesized using iScript cDNA Synthesis Kit (Bio-Rad).

Quantitative PCR (qPCR) was performed with the Applied Biosystems Viia™ 7 real-time PCR system for different tissues using a qPCR iQ SYBR Green Supermix kit (Bio-Rad) according to manufacturer's instructions and using appropriate real-time PCR protocol for *AmGAS* (RtGAS) and housekeeping genes (Table 1). Every sample was run in three parallel reactions and the amplification specificity of primers was evaluated by melting curve analysis.

The relative gene expression levels were calculated using the comparative *C*_*t*_ (Δ Δ *C*_*t*_) method (Schmittgen and Livak, 2008). According to this method, the relative gene expression is calculated as 2^−ΔΔ^ *^C^*^*t*^, where *C*_*t*_ represents the threshold cycle.

## Results

### Composition of volatile blend of *A. millefolium* flowers and leaves

The volatiles of *A. millefolium* detected in the emission blends were mostly monoterpenes (67% of total emissions for flowers and 59% of total emissions for leaves, and 21 compounds were above the detection threshold) and sesquiterpenes (17% for flowers and 19% for leaves, and 18 compounds were above the detection threshold, Table 2). In addition, lipoxygenase pathway volatiles (2% of total emissions for flowers and 11% of total emissions for leaves) and benzenoids, aliphatic compounds and their derivatives were found in minor proportions (Table 2). Among all the emitted compounds, β-pinene (36% of monoterpenes), (*E*)-β-caryophyllene and germacrene D (Table 3) were the main floral and leaf volatiles.

**Table 2:** **Relative proportions of volatiles (%) detected in the emissions of yarrow (*Achillea millefolium*) flowers and leaves**.

**Volatile**	**Retention index^a^**		**Flower mean ± SE *n* = **3****	**Leaf mean ± SE *n* = **3****
	**Lit**.	**Calc.^b^**		
**LIPOXYGENASE PATHWAY PRODUCTS**				
1-Hexanol	871	858	0.047 ± 0.038	0.67 ± 0.38
(3*Z*)-Hexenol	859	863	0.39 ± 0.26	0.84 ± 0.63
(3*Z*)-Hexenyl acetate	1005	1008	1.32 ± 0.67	8.6 ± 4.1
(3*Z*)-Hexenyl pentanoate		1237	–	1.08 ± 0.88
**MONOTERPENOIDS**				
Santolina triene	909	902	1.02 ± 0.83	1.29 ± 1.06
α-Thujene	930	926	0.309 ± 0.095	0.262 ± 0.098
α-Pinene	939	932	4.93 ± 0.73	3.31 ± 0.67
Camphene	954	949	0.28 ± 0.17	0.073 ± 0.030
Sabinene	975	975	13.5 ± 3.9	15.4 ± 8.7
β-Pinene	979	980	23.1 ± 1.9	11.9 ± 2.5
Myrcene	991	988	1.22 ± 0.34	1.33 ± 0.32
α-Phellandrene	1003	1007	0.45 ± 0.37	0.91 ± 0.30
Limonene	1029	1029	2.41 ± 0.19	2.46 ± 0.14
β-Phellandrene	1030	1033	1.19 ± 0.32	1.62 ± 0.42
1,8-Cineole	1031	1036	7.8 ± 2.2	0.79 ± 0.56
(*Z*)-β-Ocimene	1037	1035	2.92 ± 0.57	3.65 ± 1.61
(*E*)-β-Ocimene	1050	1046	5.4 ± 2.1	12.5 ± 6.9
γ-Terpinene	1060	1058	1.39 ± 0.54	1.89 ± 0.70
*cis*-Sabinene hydrate	1070	1075	0.210 ± 0.088	–
Terpinolene	1089	1085	0.121 ± 0.015	0.157 ± 0.069
*trans*-Pinocarveol	1139	1147	0.121 ± 0.099	0.155 ± 0.080
Camphor	1146	1153	0.064 ± 0.012	0.29 ± 0.14
Pinocarvone	1165	1168	0.164 ± 0.069	0.104 ± 0.051
Borneol	1169	1179	0.191 ± 0.059	0.075 ± 0.062
α-Terpineol	1189	1197	0.415 ± 0.088	0.29 ± 0.13
**SESQUITERPENES**				
δ-Elemene	1338	1340	–	0.29 ± 0.24
α-Cubebene	1351	1352	0.065 ± 0.053	–
α-Copaene	1377	1382	0.138 ± 0.031	0.311 ± 0.059
β-Bourbonene	1388	1391	0.56 ± 0.16	0.29 ± 0.11
β-Cubebene	1388	1394	0.16 ± 0.13	0.044 ± 0.036
β-Elemene	1391	1390	0.107 ± 0.087	0.44 ± 0.35
α-Isocomene	1388	1397	–	0.111 ± 0.057
(*E*)-β-Caryophyllene	1419	1428	6.7 ± 1.4	7.78 ± 3.03
*trans*-α-Bergamotene	1435	1434	1.88 ± 0.99	0.226 ± 0.052
(*Z*)-β-Farnesene	1443	1455	0.53 ± 0.32	0.51 ± 0.13
α-Humulene	1455	1464	0.71 ± 0.13	0.77 ± 0.17
*cis*-Muurola-4(14),5-diene	1467	1470	0.111 ± 0.011	0.064 ± 0.028
Germacrene D	1485	1489	4.8 ± 1.4	6.8 ± 3.4
α-Zingiberene	1494	1499	0.16 ± 0.13	–
Bicyclogermacrene	1500	1504	0.203 ± 0.055	0.26 ± 0.10
(*E*,*E*)-α-Farnesene	1506	1506	0.37 ± 0.22	0.65 ± 0.22
**SESQUITERPENES**				
Germacrene A	1509	1512	0.074 ± 0.057	0.22 ± 0.15
γ-Cadinene	1514	1521	0.057 ± 0.031	0.062 ± 0.051
δ-Amorphene	1512	1525	0.39 ± 0.22	0.41 ± 0.23
β-Sesquiphellan-drene	1523	1529	0.042 ± 0.020	0.246 ± 0.085
**OTHER VOLATILES**				
3-Methylbutanoic acid		853	1.47 ± 1.04	–
2-Methylbutanoic acid		862	0.59 ± 0.45	–
1-Nonene		893	7.7 ± 2.3	5.4 ± 3.8
Benzaldehyde	960	967	1.61 ± 0.73	–
*p*-Cymene	1025	1028	2.40 ± 1.01	1.75 ± 0.66
4,8-Dimethyl-1,3-*E*,7-nonatriene (DMNT)		1115	0.110 ± 0.079	2.49 ± 1.32
Methyl salicylate (MeSA)	1192	1198	0.101 ± 0.015	1.23 ± 0.56

a Retention indices (Adams, 2001).

b Retention indices calculated by injecting the hydrocarbon standard of C_8_ to C_20_ (Sigma-Aldrich, St. Louis, MO, USA) to GC-MS.

**Table 3:** **Relative proportions (%) of sesquiterpenes detected in the emission of flowers and leaves of yarrow (*Achillea millefolium*)**.

**Volatile**	**Retention index^a^**		**Flower mean ± SE *n* = **3****	**Leaf mean ± SE *n* = **3****
	**Lit**.	**Calc**.		
δ-Elemene	1338	1340		0.98 ± 0.81
α-Cubebene	1351	1352	0.33 ± 0.27	
α-Copaene	1377	1382	0.79 ± 0.27	1.71 ± 0.20
β-Bourbonene	1388	1391	3.18 ± 0.82	1.50 ± 0.52
β-Cubebene	1388	1394	0.82 ± 0.67	0.15 ± 0.12
β-Elemene	1391	1390	0.61 ± 0.49	4.8 ± 3.8
α-Isocomene	1388	1397		0.51 ± 0.29
(*E*)-β-Caryophyllene	1419	1428	38.7 ± 5.8	37.3 ± 14.2
*trans*-α-Bergamotene			13.0 ± 7.4	1.22 ± 0.18
(*Z*)-β-Farnesene		1455	3.8 ± 2.4	2.63 ± 0.43
α-Humulene	1455	1464	3.87 ± 0.42	4.07 ± 0.46
*cis*-Muurola-4(14),5-diene	1467	1470	0.69 ± 0.14	0.26 ± 0.11
Germacrene D	1485	1489	27.0 ± 7.1	32.4 ± 9.8
α-Zingiberene	1494	1499	0.83 ± 0.68	
Bicyclogermacrene	1500	1504	1.15 ± 0.28	1.87 ± 1.1
(*E*,*E*)-α-Farnesene	1506	1506	2.2 ± 1.2	5.1 ± 2.9
Germacrene A	1509	1512	0.42 ± 0.32	2.3 ± 1.7
γ-Cadinene	1514	1521	0.30 ± 0.16	0.21 ± 0.17
δ-Amorphene	1512	1525	2.09 ± 0.99	1.8 ± 0.6
β-Sesquiphellandrene	1523	1529	0.220 ± 0.099	1.1 ± 0.4
Total emission of sesquiterpenes			100	100

a Retention indices as in Table 2.

### Determination of germacrene a in *A. millefolium* volatile blend

**Figure 3:**
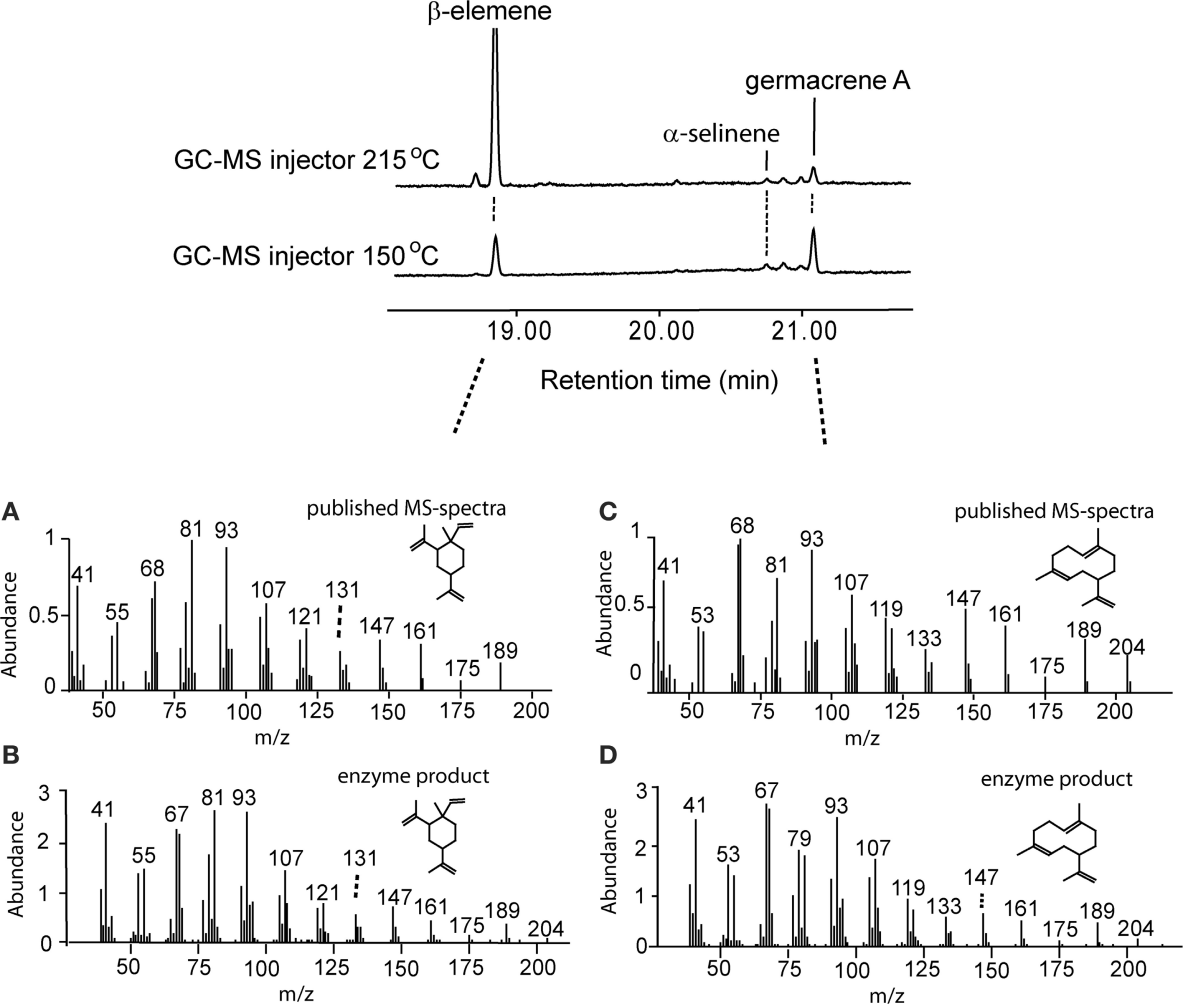
**Confirmation of the recombinant germacrene A synthase (GAS) activity of heterologously expressed GAS synthase of *A. millefolium in vitro***. Comparison of the formed product profiles using the injector temperatures of 215°C (upper chromatogram) and 150°C (lower chromatogram). Germacrene A synthase (GAS) activity was assayed with C15 farnesyl diphosphate (FDP) and the volatiles were analyzed in the headspace. Published (**A**,**B**; Adams, 2001) and observed **(B,D)** mass spectra of β-elemene **(A,B)**, and germacrene A **(C,D)** are also shown.

Germacrene A is heat-labile and is converted to β-elemene upon heating (De Kraker et al., 1998). First, we used the roots of chicory (*C. intybus*) known to contain and emit germacrene A for optimization of sampling and GC analysis protocols. Based on this work, a GC injector temperature of 150°C was used in all GC-MS analyses, resulting in a significantly increased fraction of germacrene A detected compared to β-elemene, although a large part of germacrene A was still converted to β-elemene (Figure 3). Germacrene A mass-spectrum of *in vitro* analyses of *AmGAS* with FDP as substrate matched with the published spectrum, except for differences in the proportions of mass-fragments of 79 and 81 (Figures 3C,D). In earlier studies we have noticed similar differences with the identification of some sesquiterpenes using authentic standards, e.g., identification of germacrene D, suggesting that these minor differences were specific to the GC-MS device used.

Average germacrene A emission was between ca. 0.4 and 2.3% of total sesquiterpene emission in *A. millefolium* (Table 3). Assuming further that β-elemene detected in the emission blend is the conversion product of germacrene A, the emission estimates are ca. 1% for flowers and 7% for leaves (Table 3), suggesting that germacrene A is a minor component in the volatile blend of flowers, and a moderately high component in leaf sesquiterpene emissions (Table 3).

Next to the emissions we evaluated also the chemical composition and content of terpenoids in bud, flower and leaf extracts of *A. millefolium*. We observed statistically similar amounts (10.6 ± 2.4 μg g^−1^ DW) of germacrene A in bud, flower and leaf extracts of *A. millefolium.*

### Cloning of germacrene A synthase in *A. millefolium*

*AmGAS* partial sequence was amplified by degenerate primers (green boxes in Figure 2 show position of the degenerate primers) and then the full length was obtained by 5′ and 3′ amplification of cDNA ends (RACE-PCR). The length of the coding sequence of *AmGAS* is 1680 bp, and it encodes a protein of 559 AA residues with the predicted molecular weight of 62 kD and isoelectric point (pI) of 5.24 http://web.expasy.org/compute_pi/). The overall length and lack of the characteristic chloroplast-targeting signal peptide suggests that *AmGAS* is functional in the cytosol.

The blast searches in NCBI and UniProtKB showed that *AmGAS* belongs to terpenoid synthase (TPS) gene subfamily TPS-a (Bohlmann et al., 1998), and has a high similarity with germacrene A synthases of two other members of Asteraceae, *T. parthenium* (F8UL80) and *A. annua* (I3WAC7) (Figure 1). Multiple sequence alignment of AmGAS amino acid sequences for several additional Asteraceae family members further showed conserved motifs of terpenoid synthases (Figure 2). Nevertheless, AmGAS also has a relatively high homology with other sesquiterpene synthases from Asteraceae, while the similarity is much less with other TPS-a gene subfamily members in other angiosperms (Figure 1).

### Gene cloning and expression of germacrene A synthase

**Figure 4:**
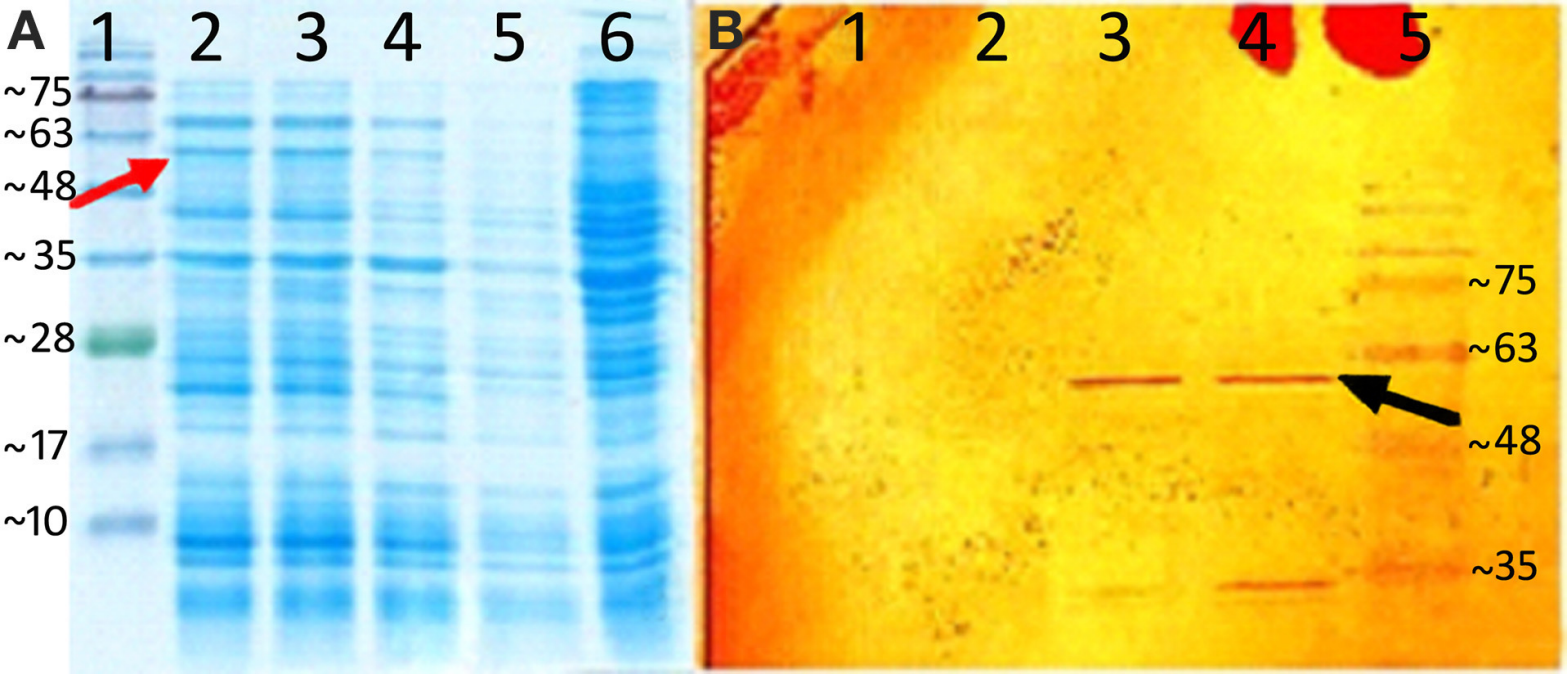
**Heterologus expression of AmGAS**. **(A)** sodium dodecyl sulfate polyacrylamide gel electrophoresis (SDS-PAGE), the numbers indicate—1: protein marker, 2: induced recombinant strain containing pET-26b (+) plus *AmGAS* gene after 6 h, 3, after 4 h; 4, after 2 h; 5, non-induced and 6, *E. coli* BL21 (DE3) without plasmid, and **(B)** western blotting, the numbers denote—1, non-induced recombinant strain containing pET-26b (+) plus *AmGAS* gene; 2, induced protein after 2 h; 3, after 4 h; 4, after 6 h and 5, protein marker. Arrows show the band that belongs to the recombinant protein of AmGAS.

Recombinant protein expression after induction was analyzed by SDS-PAGE and western blotting. Analysis of the recombinant protein expression by SDS-PAGE demonstrated a protein band at around 62 kD in induced recombinant strain samples containing pET-26b (+) plus *AmGAS*. This band corresponding to the calculated molecular mass of AmGAS protein was not observed in negative control and non-induced samples (Figure 4A). The western blotting also confirmed the expression (Figure 4B).

### Functional characterization of recombinant germacrene A synthase

Functional characterization of AmGAS *in vitro* was carried out by incubation with farnesyl diphosphate (FDP), the substrate for sesquiterpenes, and geranyl diphosphate (GDP) and neryl diphosphate (NDP), the substrates for synthesis of monoterpenes. Incubation with FDP yielded β-elemene and germacrene A as the main volatiles in the headspace with minor contributions of α- and β-selinene (Table 3, Figure 3). The percentage of germacrene A detected was greater for lower injector temperature (Figure 3), again suggesting that the bulk of β-elemene might reflect the heat conversion of germacrene A.

**Figure 5:**
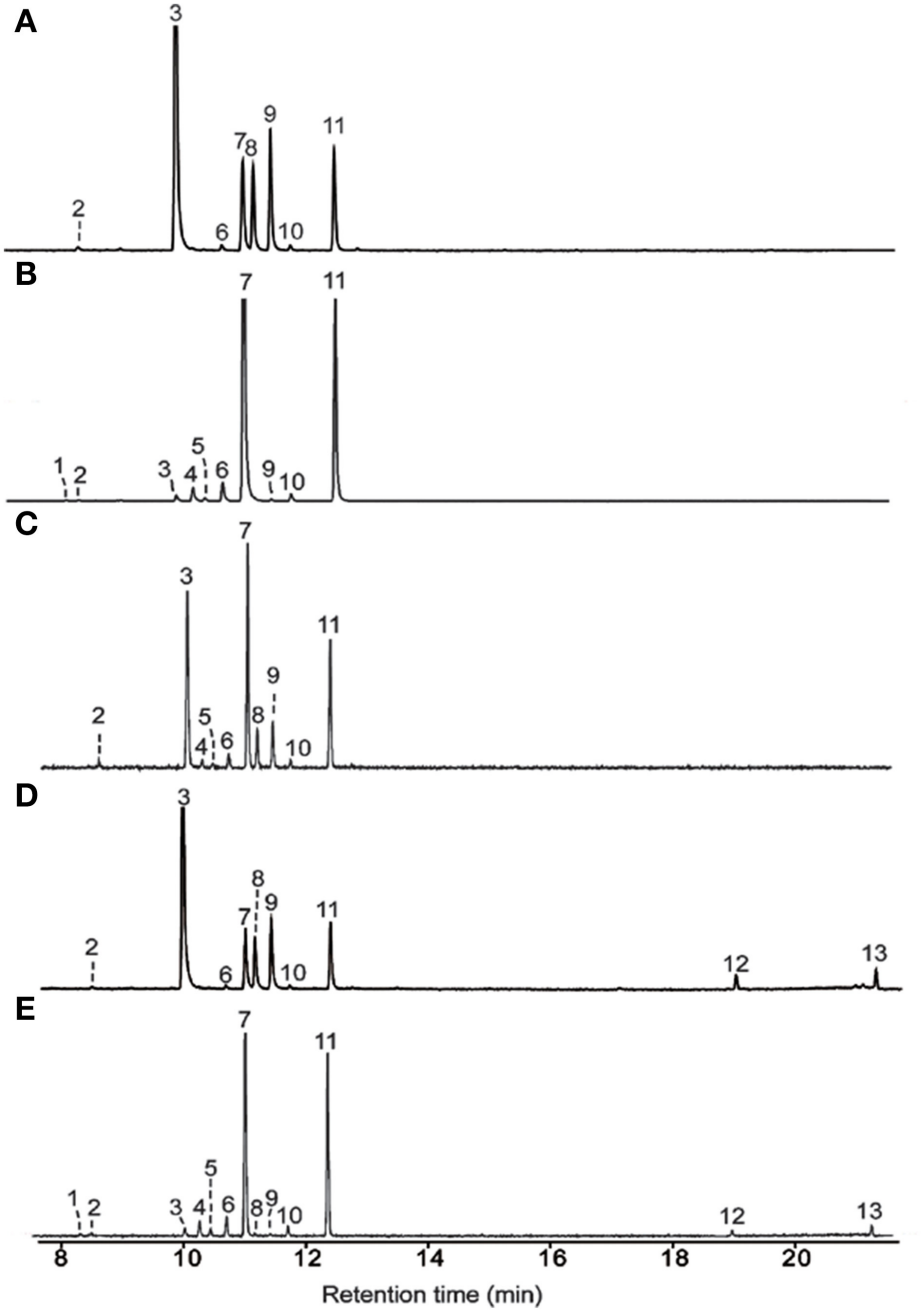
**Terpenoids detected in the vapor phase of *in vitro* analysis**. AmGAS was fed with geranyl diphosphate (GDP) **(A)**, neryl diphosphate (NDP) **(B)**, the mixture (1:1) of neryl diphosphate (NDP) and geranyl diphosphate (GDP) **(C)**, geranyl diphosphate (GDP) and farnesyl diphosphate (FDP) **(D)** or neryl diphosphate (NDP) and farnesyl diphosphate (FDP) **(E)** at gas chromatograph mass spectrometry injector temperature of 150°C. 1, α-thujene; 2, α-pinene; 3, myrcene; 4, 2-carene; 5, α-phellandrene; 6, α-terpinene; 7, limonene; 8, (Z)-β-ocimene; 9, (E)-β-ocimene; 10, γ-terpinene; 11, terpinolene; 12, β-elemene; 13, germacrene A.

Incubation of AmGAS with GDP produced mainly aliphatic monoterpenes myrcene and *Z*- and *E*-β-ocimene, but cyclic monoterpenes limonene and terpinolene were also produced at moderately high levels (Figure 5A, Table 4). AmGAS with NDP produced mainly limonene and terpinolene (Figure 5B, Table 4).

**Table 4:** **Terpenoids detected in the headspace of *in vitro* analysis of recombinant *A. millefolium* germacrene A synthase (AmGAS) fed with geranyl diphosphate (GDP), neryl diphosphate (NDP) or farnesyl diphophate (FDP) at gas chromatograph mass spectrometry (GC-MS) injector temperature of 150°C**.

**Substrate**	**Product**	**Retention index**		**Relative proportion (%)**
		**Lit**.	**Calc**.	
FDP	β-elemene	1391	1390	47.5
	β-selinene	1490	1493	4.9
	α-selinene	1498	1500	7.1
	germacrene A	1509	1512	40.5
GDP	α-thujene	926	926	<0.1
	α-pinene	939	932	1.2
	Camphene	954	949	0.2
	myrcene	991	988	51.8
	α-terpinene	1017	1016	0.6
	limonene	1029	1029	11.5
	(*Z*)-β-ocimene	1037	1035	8.8
	(*E*)-β-ocimene	1050	1046	12.2
	γ-terpinene	1060	1058	0.6
	terpinolene	1089	1085	11.6
	linalool	1097	1099	0.8
	α-terpineol	1189	1197	0.3
NDP	α-thujene	926	926	0.2
	α-pinene	939	932	0.2
	α-fenchene	953	941	<0.1
	camphene	954	949	<0.1
	myrcene	991	988	0.8
	2-carene	1002	997	2.1
	α-phellandrene	1003	1004	0.4
	α-terpinene	1017	1016	2.9
	limonene	1029	1029	62.5
	(*Z*)-β-ocimene	1037	1035	0.5
	(*E*)-β-ocimene	1050	1046	0.2
	γ-terpinene	1060	1058	1.0
	terpinolene	1089	1085	29.1
	α-terpineol	1189	1197	<0.1

When equimolar concentrations of GDP and NDP were provided, AmGAS produced only monoterpenes (Figure 5C). With GDP and FDP or NDP and FDP, AmGAS produced both mono- and sesquiterpenes, whereas monoterpene production was favored over sesquiterpene production (Figures 5D,E).

### RNA profiling of germacrene A synthase in different tissues

**Figure 6:**
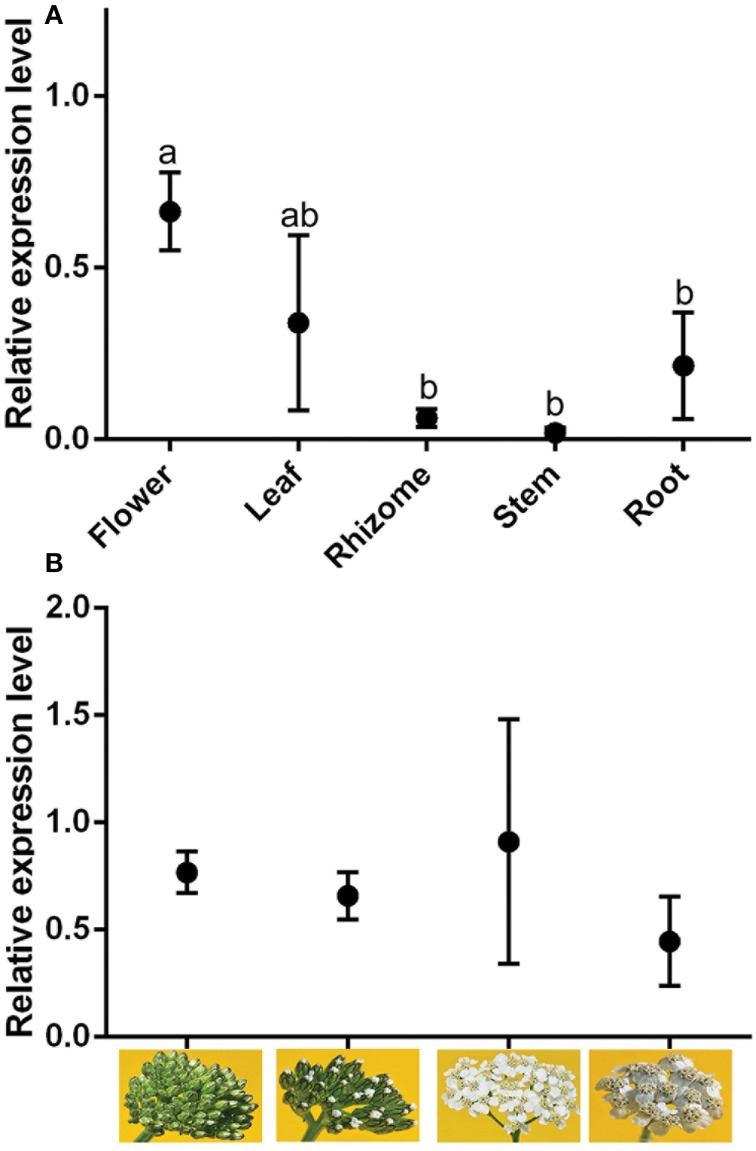
**Relative gene expression of germacrene A synthase (*GAS*) in plant**. The flower stages separated in **(B)** were from left to right: bud, early flowering, full flowering, and senescence. The expression of *GAS* is normalized with respect to the expression of glyceraldehyde 3-phosphate dehydrogenase (GAPDH) gene. Data are means ± SE. Means with the same letter in **(A)** are not statistically different (*P* < 0.05) according to paired-samples *t*-tests. No statistical differences were found for **(B)** (separate-samples *t*-tests).

Quantitative (real-time) PCR measurements of *AmGAS* were conducted with different tissues, including leaf, rhizome and root, and for flowers at different stages of development (bud, early flowering, full flowering and senescence). *AmGAS* was expressed in all organs, but the relative expression level was higher in flowers and leaves than in roots, stems and rhizomes (Figure 6A). However, flower developmental stage did not significantly alter the relative expression of *AmGAS*, although the variability was large (Figure 6B). The results were quantitatively identical by using the expression level of either β-actin or glyceraldehyde 3-phosphate dehydrogenase, the housekeeping genes selected, to normalize the *AmGAS* expression.

## Discussion

### Terpenoid volatiles in *A. millefolium* and contributions of germacrene a and β-elemene

Content and composition of secondary metabolites in *A. millefolium* tissues has been addressed in several publications (Orav et al., 2006; Gudaityte and Venskutonis, 2007; Raal et al., 2012; Dias et al., 2013). These studies have demonstrated occurrence of multiple chemotypes with varying chemical composition of the essential oil, although monoterpenes β-pinene, α-pinene, sabinene, limonene, 1,8-cineole, β-phellandrene and ocimenes and sesquiterpenes (*E*)-β-caryophyllene and germacrene D have often been observed as the main constituents of yarrow essential oil (Mockute and Judzentiene, 2003; Orav et al., 2006; Gudaityte and Venskutonis, 2007; Judzentiene and Mockute, 2010; Raal et al., 2012) agreeing with our observations (Tables 2, 3).

Germacrene A has been detected in past studies in trace level in only some chemotypes (Gudaityte and Venskutonis, 2007), and some studies have not detected germacrene A (Cornu et al., 2001; Lyakina, 2002; Orav et al., 2006; Raal et al., 2012). On the other hand, β-elemene was observed in all tested *A. millefolium* chemotypes at a significant level in the study of Gudaityte and Venskutonis (2007). However, all these past studies have used high GC injector temperatures of 230 to 250°C. Given that germacrene A is heat labile and is converted to β-elemene through the Cope rearrangement upon heating (De Kraker et al., 1998; Adio, 2009a), lack of germacrene A identification in several past studies can have resulted from excessive injector temperatures.

Here we analyzed *A. millefolium* volatiles in two different injector temperatures of 150°C and 215°C. Similarly to earlier findings (De Kraker et al., 1998), moderately high injector temperature of 215°C caused the rearrangement of the bulk of germacrene A into β-elemene, while at the injector temperature of 150°C, much greater germacrene A detection yield was achieved (Figure 3). In fact, considering that β-elemene is the rearrangement product of germacrene A, the predicted contribution of germacrene A emission to total sesquiterpene emission was ca. 1% for flowers and 7% for leaves, indicating that germacrene A is a moderately important sesquiterpene in the emissions of *A. millefolium* (Table 3). However, the sum of germacrene A and β-elemene at the injector temperature of 150°C was less than that at the injector temperature of 215°C (Figure 3), suggesting imperfect desorption at this temperature. Thus, the release of germacrene A from *A. millefolium* can be even higher than detected by the modified procedure with mild injector temperature.

### Identification of germacrene synthase gene in *A. millefolium* and RNA profiling in different tissues

Germacrene A synthase has been previously amplified in different Asteraceae family members (Figure 1) including *T. parthenium* (Majdi et al., 2011), *C. intybus* (Bouwmeester et al., 2002), *Solidago canadensis* (Prosser et al., 2002), *Helianthus annuus* (Göpfert et al., 2009, 2010), *Crepidiastrum sonchifolium* (Ren et al., 2006), *Matricaria recutita* (Irmisch et al., 2012), *Lactuca sativa* (Bennett et al., 2002), *A. annua* (Bertea et al., 2006) and *Ixeris dentata* (Kim et al., 2005). Here we further amplified the germacrene A synthase gene (*AmGAS*) from important medicinal plant *A. millefolium*, cloned and expressed *AmGAS* in *E. coli* BL21 (DE3) and confirmed the bacterial expression by SDS-PAGE and western blotting (Figure 4). Thus, a new promising plant model system was developed to investigate the regulation and evolution of germacrenes' family of sesquiterpene synthesis.

RNA profiling of *AmGAS* in *A. millefolium* tissues showed different levels of germacrene A synthase in different tissues with the highest expression level observed in leaves and flowers and much lower expression level in rhizome, root and stem tissues (Figure 6). Organ-specific expression profile of *AmGAS* is in agreement with previous observations in other species having germacrene A synthases (De Kraker et al., 2001; Bouwmeester et al., 2002; Kim et al., 2005; Nguyen et al., 2010). Nevertheless, in some plant species such as *T. parthenium* even a more specialized *GAS* expression pattern has been found with the expression mainly confined to flowers and very low expression level or none in leaves and roots (Majdi et al., 2011). Future studies are needed to gain insight into regulatory elements responsible for organ-specific expression pattern and species differences in organ-specificity of expression.

### Enzyme assay of germacrene A synthase in *A. millefolium*

AmGAS analysis with different substrates indicated that it is a multi-substrate enzyme that is capable of binding either C10 substrates GDP or NDP to form monoterpenes, or C15 substrate FDP to form sesquiterpenes (Table 4, Figures 3, 5). Although multiple substrates are not routinely used in functional characterization of terpenoid synthases, it has been demonstrated that several terpenoid synthases are capable of using multiple substrates (Rajabi et al., 2013). For example, Steele et al. (1998a) showed that sesquiterpene δ-selinene synthase and γ-humulene synthase from conifer *Abies grandis* could produce monoterpenes when incubated with GDP *in vitro*. Analogously, sweet basil (*Ocimum basilicum*) α-zingiberene synthase can catalyze formation of several cyclic monoterpenes when GDP is provided as substrate (Davidovich-Rikanati et al., 2008). In apple (*Malus domestica*) sesquiterpene α-farnesene synthase formed monoterpenes, in particular acyclic monoterpenes, *E*-β-ocimene, myrcene and linalool when GDP was given as substrate (Green et al., 2007). This latter result is analogous to AmGAS reaction with GDP where mainly acyclic monoterpenes were produced in our study (Table 4).

It is interesting that *AmGAS* incubation with NDP resulted in production of cyclic monoterpenes, while incubation with GDP mainly resulted in production of acyclic monoterpene. This indicates that substrate structure importantly drives the product profiles of AmGAS. It is plausible that the *trans*-substrate, GDP, ionizes mainly to linalyl cation, resulting in production of acyclic products, while the *cis*-substrate, NDP, ionizes to neryl cation and further to terpinyl cation leading to production of cyclic monoterpenes (Schilmiller et al., 2009). The linalyl cation can further isomerize to neryl cation, but the reverse, *cis*-*trans*-isomerization is likely sterically restricted as no acyclic monoterpenes were formed with NDP.

Despite AmGAS has the monoterpene synthase activity similarly to some other sesquiterpene synthases, the functional significance of this finding, especially the finding of the use of potential use of NDP, is not fully clear. Traditionally, monoterpene synthesis has been considered to occur in plastids, while sesquiterpene synthesis in cytosol (Dudareva et al., 2004, 2006; Pichersky et al., 2006; Tholl, 2006; Bohlmann and Keeling, 2008; Chen et al., 2011; Gutensohn et al., 2013; Rajabi et al., 2013). This understanding stems from the evidence of subcellular localization of pertinent terpenoid synthases and distribution of GDP (assumed to be mainly in chloroplasts) and FDP (assumed to be mainly in cytosol). Chloroplastic monoterpene synthases have a typical transit peptide at the N-terminal position which is responsible for chloroplast targeting. Thus they are 50–70 amino acids longer (600–650 amino acids) than sesquiterpene synthases which lack a transit peptide and contain 550–580 amino acids (Bohlmann et al., 1998; Rajabi et al., 2013). Lack of the transient peptide and overall length of AmGAS (559 amino acids), suggest that AmGAS is functionally active in the cytosol. As in our study, a greater affinity to GDP than to FDP has been observed for some other sesquiterpene synthases. For instance, a sesquiterpene synthase (LaBERS) from lavender, used GDP with a higher affinity than FDP and also produced monoterpenes, albeit with low rates (Landmann et al., 2007). It has been suggested that LaBERS has probably evolved from a monoterpene synthase by the loss of the plastidial signal peptide and by broadening its substrate spectrum.

On the other hand, there is recent evidence that multiple-substrate sesquiterpene synthases in cytosol can function as monoterpene synthases in cytosol when GDP becomes available (Davidovich-Rikanati et al., 2008; Gutensohn et al., 2013), presumably through the export of GDP from chloroplasts (Gutensohn et al., 2013). Previously, the cross-talk among chloroplastic and cytosolic isoprenoid synthesis pathways has been thought to occur at the level of C5 intermediate isopentenyl diphosphate (IDP) (Hemmerlin et al., 2003; Laule et al., 2003). However, the experimental evidence suggests that as yet unidentified IDP-transporter can also transport GDP (Bick and Lange, 2003). In fact, ^13^C-labeling suggests that chloroplast-derived GDP can be used in cytosolic sesquiterpene synthesis in chamomile (*M. recutita*), close relative of *A. millefolium*, (Adam and Zapp, 1998; Adam et al., 1999), suggesting that GDP can be available for cytosolic monoterpene synthesis in Asteraceae.

In transgenic tomato that expresses multiple-substrate sesquiterpene α-zingiberene synthase in cytosol, monoterpene synthesis in cytosol was relatively small unless chloroplastic GDP pool was strongly enhanced by overexpressing plastidic GDP synthase (Gutensohn et al., 2013). This evidence opens up an exciting opportunity that physiological conditions leading to buildup of chloroplastic GDP can enhance GDP transport to cytosol, leading to major enhancement of cytosolic monoterpene synthesis. In fact, our study indicated that AmGAS affinity to GDP is greater than to FDP as more monoterpenes were produced when both substrates were given in equimolar concentrations (Figure 5D). Clearly the substrate affinity, C10 vs. C15, can depend on multiple factors such as the concentration of metal cations and pH of the reaction medium (Green et al., 2007), but nevertheless this result suggests that the balance between sesqui- and monoterpenes can be importantly altered by GDP availability. Ocimene-type aliphatic sesquiterpenes synthesized by AmGAS when GDP is provided as substrate are classic stress-induced monoterpenes (Rodriguez-Saona et al., 2001; D' Alessandro and Turlings, 2005; Arimura et al., 2009; Copolovici et al., 2011, 2012) that in the case of some stresses are induced almost instantaneously in response to stress (Copolovici et al., 2012). Possible regulation of chemical profiles by enzyme substrate availability, FDP vs. GDP, provides a potential important control point for physiological regulation of cytosolic terpene synthesis.

There is also a long-standing enigma of how monoterpene synthesis proceeds in heterotrophic compartments of aromatic plants lacking specialized storage structures. Plastidic monoterpene synthesis, especially in leaves, is classically strongly linked to photosynthetic carbon metabolism (Niinemets et al., 2010; Li and Sharkey, 2013). In the case of aromatic plants such as *A. millefolium*, mono- and sesquiterpene contents of the essential oil are strongly correlated (Mockute and Judzentiene, 2003; Orav et al., 2006; Gudaityte and Venskutonis, 2007; Judzentiene and Mockute, 2010) and not necessarily correlated with the rate of carbon assimilation. Thus, the finding of mixed substrate specificity of AmGAS might indicate a more important role of cytosolic monoterpene synthesis in aromatic plants.

What could be the physiological significance of *cis*- vs. *trans*-isomers of substrates? Recently, a tomato monoterpene synthase has been sequenced that uses neryl diphosphate, the *cis*-isomer of GDP, as a substrate instead of GDP, to form several cyclic monoterpenes in trichomes (Schilmiller et al., 2009). Sallaud et al. (2009) further reported that a sesquiterpene synthase in tomato uses *Z,Z*-FDP instead of the usual *E,E*-FDP for the biosynthesis of type II sesquiterpenes in the trichome secretory cells. Clearly more work is needed to gain insight into the possible use of *cis*-substrates in species other than tomato.

### Phylogenetic analysis of germacrene A synthase

Multiple sequence alignment of AmGAS amino acid sequences with germacrene A synthases from other Asteraceae species showed high sequence similarity (Figure 2). The phylogenetic analysis showed a particularly close relationship between *GAS* from *A. millefolium* and *T. parthenium* and *A. annua* which is in accordance with high phylogenetic relatedness among these species (Figure 1). Germacrene A synthase from Asteraceae grouped in one single clad, which suggests a monophyletic origin of the gene. This is in agreement with the observation that occurrence of germacrene A is restricted to this family (Bouwmeester et al., 2002; Adio, 2009a; Majdi et al., 2011).

Germacrene A synthase is a two-domain, α−β-terpenoid synthase with the active center in α-domain (C-terminus, 234–558 AA) exhibiting class I terpene synthase activity (Christianson, 2008; Rajabi et al., 2013). β-domain in N-terminus (32–245 AA) has lost the catalytic activity in mono- and sesquiterpene synthases, and seems to play a role in tertiary conformation of α−β-terpenoid synthases (Christianson, 2006; Aaron and Christianson, 2010). Thus, the 5′ end of the *GAS* gene (N-terminus for the protein) shows considerable variation in gene structure and sequence which is in agreement with other two-domain, α−β-terpenoid synthase genes (Aubourg et al., 2002).

A number of conserved sequences of AmGAS with high homology to germacrene A synthase amino acids in other Asteraceae family members was detected. The second red box in Figure 2 shows the conserved aspartate-rich motif of DDxxD (DDTYD Asteraceae family, position 316–320 AA) which is conserved in all plant terpenoid synthases (Steele et al., 1998a). The occurrence of this aspartate-rich motif (DDxxD) at the catalytic site is crucial in positioning the substrate for catalysis. Another metal binding motif is located on the opposite side of the active site (Christianson, 2006). This motif, designated as NSE/DTE motif, has apparently evolved from a second aspartate-rich motif conserved in prenyl transferases, although this NSE/DTE motif is less conserved in sesquiterpene synthases. In grand fir (*A. grandis*) sesquiterpene δ-selinene and γ-humulene synthases, this motif is replaced by a second DDxxD motif (Steele et al., 1998a). Here we show that this motif is replaced by DDxxx (DDVMT) in germacrene A synthases of Asteraceae (the forth red box, position 460–464, Figure 2). This second DDxxD (or DDxxx) motif is also involved in catalysis (Steele et al., 1998b; Little and Croteau, 2002) and the formation of multiple products might be enhanced by this motif (Degenhardt et al., 2009).

In addition to these motifs, about 35 amino acids upstream of the first DDxxD motif there is a highly conserved arginine-rich, RxR (RDR in Asteraceae) motif (the first red box in Figure 2), that plays a role in the complexing of the diphosphate group after ionization of FDP (Starks et al., 1997). Also the third red box shows a conserved motif of TSA (position 416–418) that plays a substantial role in cyclization (Chang et al., 2005). High conservation of these motifs in germacrene A synthases from Asteraceae suggests that they have the same catalytic mechanism and are potentially mixed-substrate terpene synthases. Clearly further work with protein crystal structure is needed to gain insight into the catalysis of germacrene A synthases with different substrates and into the determinants of substrate specificity and product profiles with different substrates.

## Author contributions

LP participated in designing and carrying out the experiments, analyzing the data and writing the manuscript; HM contributed to designing and describing the methods, interpreting the data and writing; AK, performed GC-MS analysis; RB, supported real time PCR experiment; ÜN contributed to designing and planning the experiment, interpreting the data and writing.

### Conflict of interest statement

The authors declare that the research was conducted in the absence of any commercial or financial relationships that could be construed as a potential conflict of interest.
